# Hibernation Habitat Selection by the Threatened Chinese Softshell Turtle (*Pelodiscus sinensis*) in the Yellow River Wetlands of Northwest China: Implications for Conservation Management

**DOI:** 10.1002/ece3.70789

**Published:** 2025-01-17

**Authors:** Qingjun Zhu, Meiling Hong, Qiutong Xie, Fei Kong, Liu Lin, Hai‐tao Shi

**Affiliations:** ^1^ Ministry of Education Key Laboratory for Ecology of Tropical Islands, College of Life Sciences Hainan Normal University Haikou China; ^2^ Shaanxi Provincial Institute of Zoology Xian China

**Keywords:** aquatic hibernation, conservation, freshwater turtle, overwintering behavior, terrestrial hibernaculum

## Abstract

Hibernation is a crucial aspect of the life history of freshwater turtles inhabiting temperate regions. Therefore, understanding their hibernation habitat selection is essential for the targeted conservation of turtle species and their habitats. The Chinese softshell turtle ( *Pelodiscus sinensis* ), a medium‐sized freshwater turtle, is widely distributed in China; however, populations are rapidly declining, and threatened by habitat destruction, overfishing, and water pollution. Little is known regarding this species' habitat selection during the winter months. In 2020–2022, we equipped 22 *P. sinensis* with radio transmitters (VHF), and we successfully relocated 13 turtles, 11 of which were buried in submerged substrates and 2 buried in terrestrial soil for hibernation. In aquatic habitats, turtles preferred ponded areas formed during the dry period of the Yellow River with low water velocity and less anthropogenic disturbance. However, we found little evidence for the selection of dissolved oxygen levels. In terrestrial habitats, turtles are buried under densely vegetated soils with their dorsal carapace approximately 5 cm beneath the surface, allowing respiration through a protruded neck. Terrestrial hibernacula were close to the water, maintained more than 30% humidity throughout the winter, and were effectively protected against freezing. To the best of our knowledge, this is the first formal report of the behavior of terrestrial hibernation in softshell turtles. Our results suggest that *P. sinensis* has selectivity toward hibernation habitats with specific microenvironmental characteristics, indicating that protection of the characterized habitats provided in this study is important for the future conservation of this threatened softshell turtle species.

## Graphical abstract

This study investigates the hibernation habitat selection and overwintering environment of Chinese softshell turtle ( *Pelodiscus sinensis* ) in northwest China, and for the first time comprehensively demonstrates the selection characteristics of softshell turtles on the microenvironment of hibernation habitats. This study also formally reports the behavior of terrestrial hibernation in softshell turtles for the first time. These results provide valuable insights for the conservation of endangered softshell turtles.

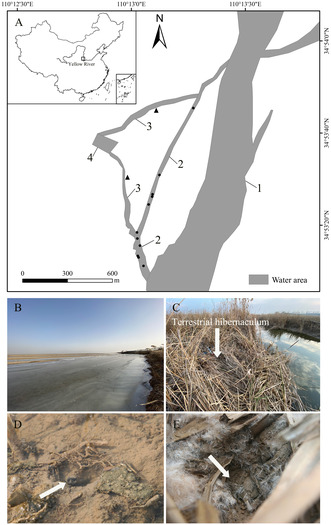

## 1 Introduction

Freshwater turtles that inhabit temperate environments have evolved mechanisms for survival in cold winters, typically by remaining dormant or inactive in long‐term stable environments (Ultsch 2006). Because hibernation is a crucial aspect of their life history, freshwater turtles in these temperate regions must seek out hibernacula with features that promote overwinter survival, including protection from risks such as hypoxia, hypothermia, and predation (Brooks, Brown, and Galbraith 1991; Brown and Brooks 1994), as well as regulating both behavioral and physiological traits (Costanzo, Lee, and Ultsch 2008), to survive extreme winter environments (Crawford 1991).

Temperature and dissolved oxygen (DO) are the most common stressors for freshwater turtles during winter (Ultsch 2006). Thus, research on hibernation habitats of freshwater turtles is often based on laboratory work on low‐temperature submergence and hypoxia tolerance (Ultsch and Jackson 1995; Jackson 2000; Reese, Jackson, and Ultsch 2002; Reese et al. 2004). Most freshwater turtles hibernate in thermally stable underwater environments where they can avoid exposure to subfreezing temperatures (Ultsch 1989). However, as a taxa that relies primarily on their lungs for respiration during the warm seasons, prolonged hibernation underwater may cause physiological stress on respiratory metabolism and, in some cases, may even be fatal (Ultsch and Jackson 1982; Jackson 2000; Jackson et al. 2000). Hence, the degree of tolerance to hypoxic environments largely determines the type of habitat that freshwater turtles use during the winter (Ultsch 2006). In general, freshwater turtles that are tolerant of hypoxic environments (e.g., *Chrysemys picta* , *Chelydra serpentina* , and *Emydoidea blandingii* ) can utilize a wide range of habitat types, including extremely anoxic wetlands (Jackson 2000; Reese, Jackson, and Ultsch 2002; Edge et al. 2009); however, species that are intolerant of anoxic conditions (e.g., *Apalone spinifera* , *Sternotherus odoratus* , and *Graptemys geographica* ) typically hibernate in well‐oxygenated microenvironments (Ultsch and Jackson 1995; Reese et al. 2001; Reese, Jackson, and Ultsch 2003).

The family Trionychidae (softshell turtles) is an extreme example of an anoxia‐intolerant freshwater turtle taxon. They are probably the least anoxia‐tolerant group of freshwater turtles (Reese, Jackson, and Ultsch 2003); however, their oropharyngeal cavities have well‐developed, vascularized villous projections that can effectively take up DO from water (Stone, Dobie, and Henry 1992; Davenport and Wong 1992; Bagatto and Henry 1999). The combination of hypoxia intolerance and efficient DO uptake in softshell turtles suggests that they may hibernate in submerged environments with high oxygen levels (Reese, Jackson, and Ultsch 2003; Plummer and O'Neal 2019). Validation of these laboratory‐based results through field studies is clearly important. However, winter habitats of softshell turtles in the field are largely undocumented despite their global geographical distribution. Limited field studies have reported that they hibernate buried in the substrate at the bottom of the water (Plummer and Burnley 1997; Galois et al. 2002). There have also been anecdotal reports of softshell turtles leaving the aquatic environment in winter and burying themselves in mud (Newman 1906; Cagle 1942); however, these cases are considered atypical (Ultsch 2006). Ambiguous information concerning hibernation habitats makes the conservation of softshell turtles challenging.

The Chinese softshell turtle ( *Pelodiscus sinensis* ) is a medium‐sized freshwater turtle that is widely distributed from southern to northern China (22.4°–41.4° N) (Kong et al. 2022). In the Yellow River basin in northwest China, *P. sinensis* depends on wetland habitats, such as the main river channels of the Yellow River, associated tributaries, and ponds. These habitats are often near areas of human activity; therefore, they are negatively impacted by overfishing, habitat destruction, and water pollution (Zhu, Kong, and Shi 2021). In 2000, the species was listed as Vulnerable (VU) on the IUCN Red List (TTWG 2021). Since then, its populations have declined further, with more than 95% of wild populations disappearing (Kong et al. 2022). It has been reported that *P. sinensis* prefers areas with moderate water depths and slow water velocity in the warm seasons (Kong 2020) and this turtle species may have a low tolerance to salinity and pH (Yang, Niu, and Sun 1999). However, the habitat of *P. sinensis* in winter has never previously been reported.

The purposes of this field study were to determine the habitat types of hibernating *P. sinensis* (e.g., aquatic and/or terrestrial habitats) and how its hibernation habitat selection was influenced by environmental variables. Building on previous studies on the behavior and physiological characteristics of softshell turtles in winter (Plummer and Burnley 1997; Jackson et al. 2000; Ultsch 2006), as well as the habitat characteristics of *P. sinensis* during the warm seasons (Yang, Niu, and Sun 1999; Kong 2020), we hypothesized that (i) *P. sinensis* hibernates underwater to avoid the adverse effects of low temperatures, but the possibility of terrestrial hibernation cannot be ruled out; (ii) the selection of aquatic hibernation habitat is influenced by the physical and chemical properties of the water (e.g., depth of water, water velocity, pH, salinity, and especially the DO levels) and anthropogenic disturbances; and (iii) the aquatic hibernacula of *P. sinensis* are characterized by high oxygen levels throughout the winter.

## 2 Materials and Methods

### 2.1 Study Area

**FIGURE 1:**
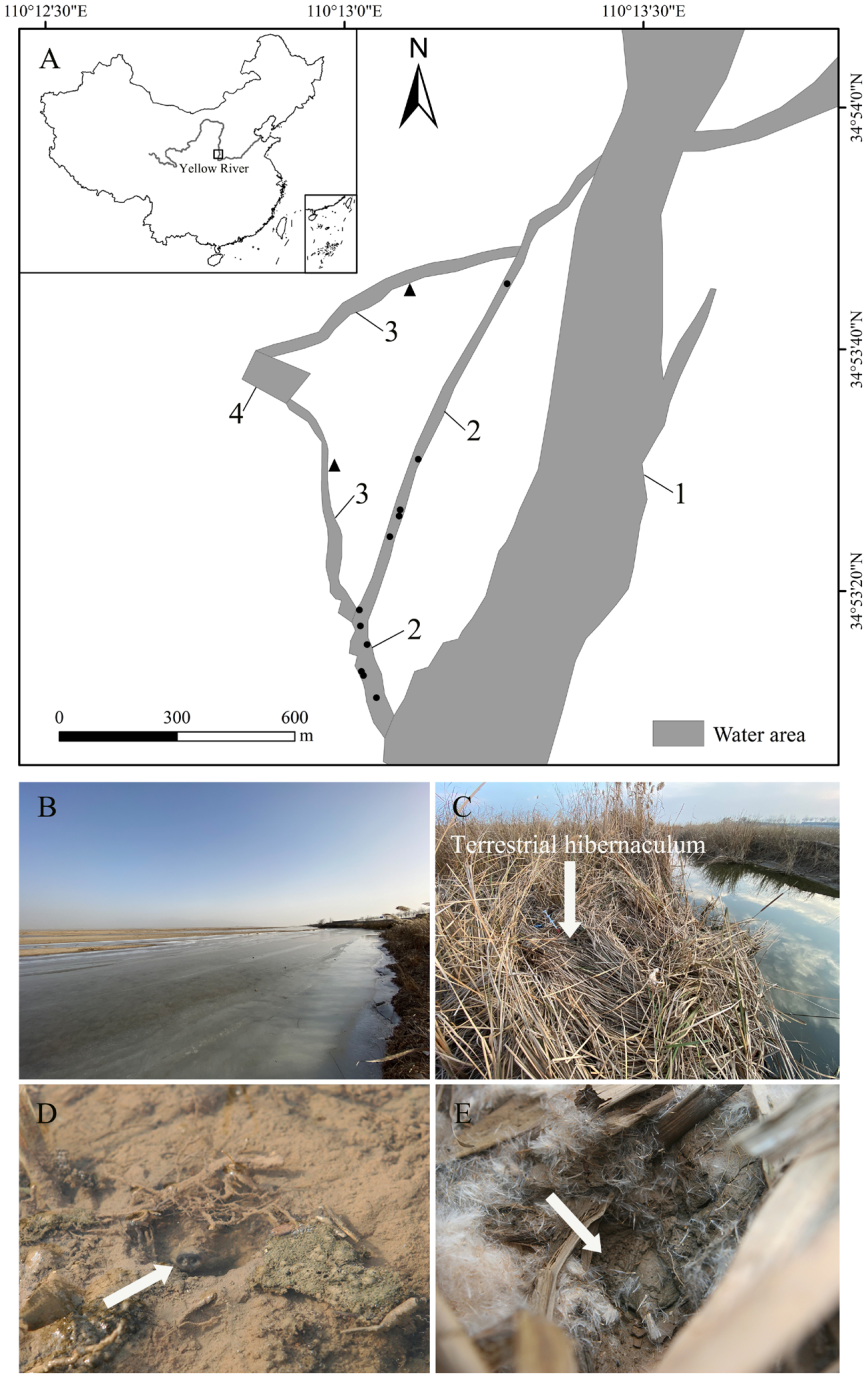
Map of the study area in Dali County, Shaanxi Province, China. Four types of aquatic habitats with typical characteristics in the study area during winter are shown by the numbers from 1 to 4, which are the Yellow River channel (YR), the ponded area (PA), the tributary (TR), and the sand mining pool (SP), respectively. The aquatic hibernating sites are shown by black dots and the terrestrial hibernating sites by triangles (A). Photos of the ponded area for aquatic hibernation (B) and the terrestrial hibernaculum in Year 1 (C). In the aquatic habitat, the turtle was completely buried in the submerged substrate but had access to the water column at the snout end (D). In the terrestrial habitat, the turtle was completely buried in the mud but had access to the air at the snout end by extending and retracting their necks (E). The two similar burial postures between aquatic and terrestrial hibernation indicated that the turtles rely on buccopharyngeal respiration while hibernating.

This study was conducted from October 2020 to April 2021 (year 1) and from October 2021 to March 2022 (year 2) in Dali County, Shaanxi Province, China (34.89° N, 110.22° E). Dali County has a continental monsoon climate with long and dry winters. Extreme low temperatures usually occur in January, with an average minimum air temperature of −9°C over the past 10 years (2015–2024, data obtained from the local meteorological bureau). The study area contains various types of aquatic habitats for *P. sinensis* , including the Yellow River, the ponded area, the tributary, and the sand mining pool (hereafter abbreviated as YR, PA, TR, and SP, respectively; Figure 1A; Table 1). These four types of habitats are interconnected, and each of them contains sediments at the bottom of the water deep enough for turtle hibernating. The vegetation in the area has been recorded in the previous study (Kong et al. 2021), dominated by *Phragmites australis* , *Acorus calamus* , and *Carex dispalata*.

**TABLE 1:** Definitions used to classify aquatic habitats in the study area.

Aquatic habitat	Description	Area in study site (ha)	Abbreviation
Yellow River	Main channel of the Yellow River, width of 200–300 m, fast‐flowing water	> 58	YR
Ponded area	Mainly the area formed on the left side of the Yellow River course after the winter drop in water level has exposed the riverbed in areas where the water level was previously shallow, width of 10–30 m, slow or static water	4.8	PA
Tributary	Extending from the ponded area, both sides of the riverbanks are densely covered by 2.5 m high *Acorus calamus* , width of 5–15 m, gentle‐flowing water	3.7	TR
Sand mining pool	A deep pit formed by man‐made sand mining, the Yellow River water flows into it through the tributary, width of 80–200 m, still water	1.2	SP

### 2.2 Telemetry

Turtles used for radiotelemetry were captured in cage‐type turtle traps in the Yellow River during the summer. In Year 1, eight wild turtles were tagged with radio transmitters (164 MHz; AL‐2F; Holohil Systems Ltd., Canada; see Kong et al. 2021, for more information on the method of attachment of the transmitters) and were released at the capture sites. Turtles were tracked daily beginning in early October of each year using a handheld receiver (TRX‐1000S, 216.000–216.999 MHz; Wildlife Materials International Inc., USA) with a three‐component folding antenna (Wildlife Materials International Inc., USA). In Year 1, the telemetry of two turtles failed, probably because they were captured by local fishermen. In Year 2, an additional 14 turtles were added to the radiotelemetry program, and 7 turtles were lost during that year.

### 2.3 Sampling of Environmental Variables for Aquatic Hibernation

Starting in early November, the water temperature in the study area generally dropped below 13°C, leading to a gradual decrease in the intensity of turtle activity. When the turtle stayed stationary at a site, we considered it to be hibernating. We immediately measured the water depth (cm) at the hibernation site, water velocity (m/s) using a flow meter (LS1206B; Fengtu Electronics Corporation Ltd., China), and DO (mg/L), pH, and salinity (ppt) approximately 50 cm below the water surface using a multifunctional water quality detector (U‐5000; HORIBA Corporation Ltd., Japan). The human population within a 10 × 10 m area centered on the hibernation site (primarily anglers and tourists) was recorded as an indicator of the effects of human disturbance on hibernation habitat selection. At the same time, we randomly selected control sites within 10–400 m of each hibernation site in a random direction (400 m is approximately the average turtle's home range, Kong et al. 2021) and measured the six environmental variables described above at the control sites in the same way. If a random site was not located in the aquatic habitat, it was repositioned. Each hibernation site corresponded to three to four control sites to cover all aquatic environments in the study area as much as possible.

When turtles hibernated, we no longer tracked them daily but visited their hibernacula every 7–12 days. All turtles were radiotracked at each visit to determine whether they had dispersed. Each visit occurred at approximately 11 a.m., and the water DO levels in each hibernation site and the corresponding control sites were measured. As all hibernation sites and control sites were distributed among the four types of habitats (Figure 1; Table 1) and the differences in DO between sites within the same habitat were not significant, data from only one random site in each habitat were used for subsequent analyses of DO differences between habitats in the hibernation period.

### 2.4 Terrestrial Hibernation Habitat

When turtles hibernated on land, we recorded the distance between their carapace and the ground surface. Since quantitative studies of habitat selection were difficult with only two terrestrial overwintering sites in this study, we only described typical characteristics of terrestrial hibernacula and recorded changes in temperature and humidity throughout the winter. In both years, one thermometer (HOBO U23‐001A; Onset Computer Corporation Ltd., USA) was suspended 1.5 m above the ground to measure air temperature at 1 h intervals. In Year 1, one thermometer (HOBO U23‐001A) was deployed at a depth of 5 cm in the soil in the terrestrial hibernaculum to measure temperatures experienced by the turtle at 3 h intervals. In Year 2, to better account for the temperatures experienced by the overwintering individual, four thermometers (GSP‐6; Jingchuang Electronics Corporation Ltd., China) were deployed uniformly at different depths in the hibernaculum (surface, 5, 10, and 15 cm), covering the maximum depths to which an individual's plastron could reach. During each visit to the terrestrial hibernaculum in Year 2, we measured humidity using a soil moisture meter (NB‐IOT; Jingxun Electronics Corporation Ltd., China). After the tracking study was completed, radiotracking transmitters were removed from the carapaces of the individuals that could be recaptured.

### 2.5 Data Analysis

All data analyses were performed using R version 4.4. We used a generalized linear model (GLM; family = binomial; logit link) to relate environmental variables to aquatic habitat selection preferences with the R package “glumlti.” The response variables were specified as either the environmental variables at the 11 hibernation sites (1) or 42 randomly generated sampling sites (0). Model selection procedures were used to evaluate the strength of evidence for the relative influence of the different independent variables included in the models. The full models included all six environmental variables. All possible combinations of variables (hereafter “candidate model”) were then derived from the full models. We used information‐theoretic multimodel inference based on Akaike's information criterion (Akaike 1973) corrected for small sample sizes (Hurvich and Tsai 1989) to rank the AICc differences (hereafter “Δ_*i*_”) between the candidate models from best to worst. Models within 2.0 units (Δ_*i*_ < 2) of the optimal model were considered as having support. To further compare the models, we calculated the Akaike weights (i.e., the relative support for the alternative models, Burnham and Anderson 2002) of each model and reported the confidence set of all candidate models ranked downward from the AICc best model with a sum of weights equal to 0.95 (Whittingham et al. 2005). The relative importance of a variable (*W*_+_) was estimated by adding the AICc weights across all candidate models in which the variable occurred. The model‐averaged parameter estimates (*β*) and the estimate precision (unconditional standard errors, SE) were calculated using the R package “MuMIn”. The *W*_+_ and *β* values inform us of the strength of importance of each variable (Burnham and Anderson 2002).

In addition, we used paired *t*‐tests to analyze the differences in DO between ice cover and non‐ice cover periods of the four types of aquatic habitats in the study area (Table 1), as well as between selected and unselected habitats (Figure 1). The Kolmogorov–Smirnov test was used to test the normality of the data before the analysis. Descriptive statistics are expressed as mean ± standard deviation (SD). The significance level was set at *p* < 0.05.

## 3 Results

### 3.1 Habitat Selection in Aquatic Hibernation

In total, telemetry data were obtained for 13 individuals over 2 years. Of these, 11 turtles hibernated in aquatic habitats and 2 in terrestrial habitats (Table 2). In the aquatic habitats, all turtles hibernated only in habitat PA in both years (Figure 1A), although they were present in other types of habitats during their search for overwintering habitats before hibernation. Underwater overwintering turtles entered hibernation as early as November 23 and as late as December 10; they ended hibernation as early as March 10 and as late as April 3. All turtles stayed stationary throughout the winter.

**TABLE 2:** Information of 22 Chinese softshell turtles ( *Pelodiscus sinensis* ) radiotracked from October 2020 to April 2022.

No.	Sex	Weight (g)	Carapace length (cm)	Hibernation type
Y1‐314	Female	960	19.3	Aquatic hibernation
Y1‐092	Male	1160	21.5	Aquatic hibernation
Y1‐277	Female	1217	21.8	Aquatic hibernation
Y1‐357	Male	1036	20.2	Aquatic hibernation
Y1‐334	Female	1049	19.9	—
Y1‐342	Female	1166	20.4	Terrestrial hibernation
Y1‐258	Female	1168	21.6	Aquatic hibernation
Y1‐176	Female	1394	22.2	—
Y2‐926	Female	1237	21.7	—
Y2‐590	Male	1242	21.1	Aquatic hibernation
Y2‐860	Male	918	20	—
Y2‐125	Male	1499	22.8	Terrestrial hibernation
Y2‐492	Female	1329	21.7	—
Y2‐442	Female	1282	20.9	Aquatic hibernation
Y2‐342	Male	1229	24	Aquatic hibernation
Y2‐409	Female	1297	22.1	—
Y2‐026	Female	1203	20.8	Aquatic hibernation
Y2‐393	Female	1240	21.5	Aquatic hibernation
Y2‐075	Female	1430	22.5	Aquatic hibernation
Y2‐241	Male	1192	21.3	—
Y2‐210	Male	1387	23	—
Y2‐142	Male	1458	23	—

*Note:* Y1‐342 was dead during hibernation.

The model selection process based on AICc showed that the 95% confidence set of models included 16 models, of which four models (Δ_*i*_ < 2) could be considered as having support related to habitat preference (Table 3). The four supported models included all six variables, with the ranking of the coefficients being water velocity > number of humans > salinity > pH > water depth > DO. The model selection process provided strong support for the influence of water velocity and number of humans on the selection of hibernation habitats for *P. sinensis* (*W*_+_ > 0.98 and *W*_+_ = 0.92, respectively). The negative coefficients of the selection for both variables suggested that *P. sinensis* preferred to hibernate at sites with low water velocity and less anthropogenic disturbance. However, there was equivocal support for the effects of other environmental variables in our second hypothesis (Table 3), especially weak support for the water depth and DO (*W*_+_ = 0.32 and *W*_+_ = 0.23, respectively).

**TABLE 3:** Set of models for the correlation between environmental variables and habitat preferences in aquatic habitats of *Pelodiscus sinensis* .

Variable	Depth of water	Water velocity	Dissolved oxygen	Salinity	pH	Number of humans	Log Likelihood	AICc	Δ_i_	AICc weight
AIC best		1		1		1	−9.11	27.04	0	0.22
		1			1	1	−9.60	28.04	0.99	0.14
		1		1	1	1	−8.50	28.29	1.24	0.12
	1	1			1	1	−8.77	28.81	1.76	0.09
		1	1	1		1	−9.06	29.39	2.34	0.07
	1	1		1		1	−9.10	29.48	2.44	0.07
		1	1		1	1	−9.55	30.39	3.34	0.04
	1	1		1	1	1	−8.33	30.48	3.43	0.04
		1	1	1	1	1	−8.50	30.82	3.77	0.03
		1				1	−12.24	30.96	3.92	0.03
	1	1	1		1	1	−8.71	31.25	4.21	0.03
	1	1	1	1		1	−9.05	31.92	4.88	0.02
	1	1		1			−11.65	32.13	5.09	0.02
	1	1				1	−11.90	32.62	5.58	0.01
		1	1			1	−12.04	32.91	5.87	0.01
Full model	1	1	1	1	1	1	−8.30	33.09	6.04	0.01
*W*_+_	0.32	> 0.98	0.23	0.62	0.51	0.93				
*β*	−0.004	−26.432	0.013	−7.002	−4.454	−1.500				
SE	0.012	13.370	0.593	7.515	6.166	0.968				

*Note:* The models shown represent the 95% confidence set for the dataset along with the respective support (AICc weight) of each model. The variables included in the model are represented by the number. The relative importance of a variable (*W*_+_) was estimated by summing the AICc weights across all candidate models in which the variable occurred. Parameter estimates (*β*) are presented that were generated by averaging across all models (weighted by the selection probabilities).

### 3.2 Overwintering Environment in Aquatic Hibernation

During the hibernation period, ice formed on the surface of the water in all aquatic habitats, except the Yellow River, and the thickness of the ice ranged from 2 to 8 cm. Turtles buried underwater at depths of 50–120 cm; therefore, the aquatic hibernacula of *P. sinensis* were not adversely affected by ice formation throughout the winter. During the initial stage of hibernation, when the water surface was not yet frozen, DO levels were high (> 10 mg/L) in all aquatic habitats, and there were no significant differences in DO between habitats (Table 4). However, when the water surface was completely frozen, the DO levels significantly decreased in habitats TR and SP, which had no overwintering turtles. Compared with other habitats, the DO of the selected habitat PA fluctuated less throughout the winter and increased during the ice cover period (Table 4), providing strong support for our third hypothesis.

**TABLE 4:** Results of dissolved oxygen measurements (mg/L) in four types of aquatic habitats (the Yellow River, YR; the ponded area, PA; the tributary, TR; and the sand mining pool, PA) within the study area throughout the hibernation period.

Habitat	Hibernation period	Non‐ice cover period	Ice cover period	Comparison between ice cover period and non‐ice cover period
PA	11.35 ± 0.85	10.89 ± 0.40	12.00 ± 0.92	*t* = −2.875; *p* = 0.017
TR	10.47 ± 1.36	11.40 ± 0.91	9.16 ± 0.47	*t* = 5.592; *p* < 0.001
SP	10.04 ± 1.29	10.93 ± 0.84	8.79 ± 0.40	*t* = 5.191; *p* < 0.001
YR	11.19 ± 0.79			
PA vs. TR	*t* = 1.898; *p* = 0.071	*t* = −1.376; *p* = 0.205	*t* = 6.162; *p* < 0.001	
PA vs. SP	*t* = 2.934; *p* = 0.008	*t* = −0.117; *p* = 0.909	*t* = 7.149; *p* < 0.001	
PA vs. YR	*t* = 0.463; *p* = 0.648			

*Note:* The Yellow River does not freeze throughout the winter.

### 3.3 Habitat in Terrestrial Hibernation

Two terrestrial hibernacula, 75 and 60 cm away from the water, were characterized by a dense canopy of *A. calamus* , which wilts seasonally in the winter (Figure 1C). The dorsal carapaces of both turtles were approximately 5 cm beneath the surface. The hibernating turtle (No. Y1‐342) in Year 1 was found dead on December 12 (the possible reason is that the dense vegetation concealed the hibernaculum (Figure 1C), leading us to inadvertently trample on it during our initial search for its location). In Year 1, the lowest air temperature in the study area was −13.24°C, and the lowest temperature recorded in Dali County in the past 10 years (2015–2024, data obtained from the local meteorological bureau). Temperatures at a depth of 5 cm in the terrestrial hibernaculum were consistently maintained above −0.4°C, with subfreezing temperatures lasting for approximately 17 days (January 7–January 24).

The terrestrial hibernating turtle (No. Y2‐125) in Year 2 emerged from the water, entered hibernation on November 3, and returned to the water again on March 20. In Year 2, the study area experienced a more representative mild winter, with a minimum air temperature of −7.84°C. Although soil surface temperatures were consistently subfreezing, temperatures in the area where the turtle's body was located (5–15 cm) remained above freezing throughout the winter. Soil moisture in the terrestrial habitat during Year 2 was maintained at 30%–55% throughout the winter.

## 4 Discussion

In the present study, *P. sinensis* spent approximately 4 months of the year in either underwater substrates or terrestrial soil. During hibernation, it essentially stayed stationary throughout the winter, a phenomenon that has also been observed in another species of softshell turtle ( *A. spinifera* ; Plummer and Burnley 1997). Contrary to the behavior of some hard‐shelled turtles, such as *E. blandingii* and *Trachemys* spp., which move and bask in temperatures as low as 4°C to adjust body temperatures and respiration (Plummer and Burnley 1997; Sajwaj and Lang 2000; Newton and Herman 2009), softshell turtles are completely stationary in the winter, demonstrating the importance of finding suitable locations to survive from prolonged winters.

Our results suggest that water velocity is a key microenvironmental factor influencing aquatic hibernating habitat selection in *P. sinensis* . Water velocity can affect the degree of reliance on aquatic respiration in turtles (Gordos, Franklin, and Limpus 2004). Water with strong fluidity tends to have higher oxygen levels, and softshell turtles that rely more on aquatic respiration in winter can thus reduce their activity frequency (Plummer and O'Neal 2019). Previous laboratory studies of physiological characteristics and anecdotes observation have led to the inference that softshell turtles should overwinter in flowing waters because they are intolerant of anoxia (Reese, Jackson, and Ultsch 2003; Plummer and O'Neal 2019). Contrary to this prediction and observation on the habitat of softshell turtles (Graham and Graham 1997; Galois et al. 2002; Tornabene et al. 2017), *P. sinensis* preferred to hibernate in lentic waters. Specifically, hibernating *P. sinensis* was found only in the ponded area with slow water flow or stagnant water (Figure 1), but where the oxygen content of the water remained consistently above 10 mg/L throughout the winter (Table 4). The preference for areas with slow water velocity may explain why no turtles hibernated in habitat YR, where flow velocities were faster throughout the winter. The findings of this study point out a new direction for the future conservation of *P. sinensis* , that is, habitat protection should mainly involve the ponded areas formed by the Yellow River during the dry season in winter, where although the water is lentic or slow‐flowing, the oxygen content is still high.

Nevertheless, there is little evidence for the selection of DO in the GLM based on environmental variables at the hibernating and control sites, which provides limited support for our hypothesis that the selection of hibernation habitats for *P. sinensis* is influenced by oxygen levels. For softshell turtles that are intolerant to anoxia, long‐term underwater hibernation requires the selection of well‐oxygenated habitats (Reese, Jackson, and Ultsch 2003; Plummer and O'Neal 2019). A plausible reason for the lack of selectivity for oxygen levels in *P. sinensis* is that the DO levels were relatively high in each habitat during the initial hibernation period (Table 4). However, oxygen levels in aquatic habitats fluctuate constantly and are inevitably affected by ice cover (Newton and Herman 2009; present study). We found evidence of fluctuating oxygen levels in the water column within the study area, particularly in waters where no individuals overwintered (Table 4). Overall, hibernating turtles were only found in the aquatic habitat PA which did not show significant decreases in DO levels during the ice cover period. Hence, an intriguing question is whether turtles abandon some areas (e.g., habitat TR and SP in the present study) in winter because of predictable fluctuations in oxygen levels (Edge et al. 2009; Newton and Herman 2009); alternatively, they return annually to locations with specific environmental conditions for overwintering, as previously suggested (Brown and Brooks 1994; Litzgus et al. 1999; Smith and Cherry 2016). Unfortunately, our results cannot provide data support for these two hypotheses. To demonstrate the habitat selection strategy of softshell turtles more directly, it is necessary to conduct long‐term integrated monitoring of their habitats and overwintering environments, to investigate how they respond to changing environments.

Another method of obtaining adequate oxygen is to hibernate on land, where turtles have direct access to atmospheric oxygen (Buhlmann and Gibbons 2001). There have been previous reports about softshell turtles leaving the aquatic environment in the winter and burying themselves in mud (Newman 1906; Cagle 1942); however, the behavior is considered atypical, or the individuals are considered unhealthy (Ultsch 2006). Our observations do not support this opinion. In Year 2, the terrestrial hibernating individual survived the winter (> 4 months) and returned to the water again in the spring, indicating that *P. sinensis* can utilize terrestrial hibernation in northwest China. In addition, the hibernation posture that the turtle completely buried in the mud but had access to the air at the snout end by extending and retracting its neck (Figure 1E), also indicates that *P. sinensis* can fully utilize atmospheric oxygen when overwintering on land. To the best of our knowledge, this is the first formal report of the behavior of terrestrial hibernation in softshell turtles. However, due to the difficulties of field studies in softshell turtles and the low probability of occurrence of terrestrial hibernation, this study provides limited information on the terrestrial hibernacula of *P. sinensis* . Additional research on the overwintering ecology of *P. sinensis* or other softshell turtle species is necessary to provide insight into the factors contributing to the selection of terrestrial habitats. Specifically, comparative studies of the overwintering habitats of different populations of the species distributed in the north and south should be carried out, as previous studies have suggested that terrestrial hibernating occurs more often in the southern regions where winters are milder (Ultsch 2006).

Freshwater turtles that hibernate terrestrially face challenges associated with leaving water, such as the risk of freezing or dehydration (Brown and Brooks 1994; Buhlmann and Gibbons 2001; Ultsch 2006). Our results showed that the terrestrial hibernaculum of *P. sinensis* effectively prevents dehydration throughout the winter. Freezing is also not a limiting factor for turtles overwintering on land. Considering that in Year 1, Dali County experienced the coldest winter in nearly a decade, temperatures at a depth of 5 cm in the terrestrial hibernaculum were consistently maintained above −0.4°C, and plastral temperatures were predicted to be significantly higher than −0.4°C in the overwintering individual. Although the turtle that overwintered on land in Year 1 died, the death occurred before consistent subfreezing temperatures, suggesting that trampling, rather than cold temperatures, was responsible for the failure in overwintering. Soil temperatures in terrestrial habitats were consistently above freezing in Year 2 when winter temperatures were more representative. We suggest that the ability of terrestrial habitats to effectively avoid the threats of freezing and dehydration is the key to the successful overwintering of *P. sinensis* .

Additionally, habitats that can protect turtles from harsh winters must contain various features, of which the ability to avoid predation is important (Brooks, Brown, and Galbraith 1991; Brown and Brooks 1994). In the study area, *P. sinensis* populations are threatened by anthropogenic overfishing (Kong et al. 2022); therefore, we hypothesized that hibernation habitat selection of *P. sinensis* is influenced by anthropogenic disturbances. We found that, in aquatic habitats, *P. sinensis* preferred areas with less anthropogenic disturbance, which supports our hypothesis to a certain extent. In addition, the characteristics of the terrestrial hibernacula of *P. sinensis* also support our hypothesis. Both terrestrial hibernation habitats in the present study were characterized by a dense canopy of *A. calamus* . The dense vegetation covers concealed turtles coming onto land and therefore protects hibernating individuals. In both years, dense vegetation made it difficult to locate the exact locations of hibernating turtles, even with the help of radiotracking, which was a major factor that led us to trample on the terrestrial hibernating individual in Year 1.

In conclusion, we found that *P. sinensis* in northwest China can utilize aquatic and terrestrial habitats for overwintering. Their hibernacula are restricted to areas with specific environmental characteristics (Figure 1A), indicating that suitable aquatic habitats may be a limited resource in the study area. It is important to identify additional suitable habitats based on the valuable information provided in this study. Candidate sites should be evaluated for conservation opportunities for *P. sinensis* through long‐term monitoring. Notably, softshell turtles were thought to be highly dependent on aquatic environments and only come ashore for basking or egg‐laying (Yang, Niu, and Sun 1999; Kong 2020). Our results suggest that some *P. sinensis* individuals in northwestern China could spend one‐third of their lives on land. This novel finding provides a new direction for conserving this turtle species by improving protection on terrestrial habitats in winter, as turtles that hibernate on land are particularly vulnerable to disturbance and habitat destruction. Finally, *P. sinensis* may have similar habitat selection strategies and overwintering environments as other softshell turtles distributed in temperate regions, as they share some behavioral and physiological characteristics (Plummer and Burnley 1997; Bagatto and Henry 1999; Jackson et al. 2000; Plummer and O'Neal 2019). Therefore, our findings also provide a reference for the conservation of other endangered softshell turtles.

## Author Contributions

**Qingjun Zhu:** conceptualization (equal), data curation (equal), formal analysis (equal), investigation (equal), writing – original draft (equal). **Meiling Hong:** methodology (equal), supervision (equal), writing – review and editing (equal). **Qiutong Xie:** formal analysis (equal), writing – original draft (equal). **Fei Kong:** data curation (equal), writing – review and editing (equal). **Liu Lin:** conceptualization (equal), methodology (equal), supervision (equal), writing – review and editing (equal). **Hai‐tao Shi:** conceptualization (equal), funding acquisition (equal), methodology (equal), project administration (equal), supervision (equal), writing – review and editing (equal).

## Ethics Statement

This work was carried out under the ethics permit Animal Protection Society of Hainan Normal University HNECEE‐2016‐005.

## Conflicts of Interest

The authors declare no conflicts of interest.

## Data Availability

Data are available from the Dryad Digital Repository: https://doi.org/10.5061/dryad.c2fqz61js.
